# Three More within the Fold

**Published:** 1897-03

**Authors:** 


### THREE MORE WITHIN THE FOLD.

SURELY the veterinary profession over the land has no disposition to look backward, though the times have not been auspicious and the readjustment of changed forces and conditions have come with peculiar force upon its devotees. But with that earnest confidence born of the realization that in unity there is power and strength, and that our future field of labor must be broadened and enhanced in worth to our people, they have taken fresh courage; and Minnesota, on the North, is followed by Tennessee and North Carolina, on the South, in rearing State associations which are destined to be of much import in these commonwealths. Des Moines and Buffalo, in their excellent meetings of the national organization, have proven strong incentives to the establishing of State and local organizations, and much good will be felt in their birth —much added power in their deeper interest in the parent organization. Let the good work go on; let Kentucky put on her association armor and Louisiana take up the chain and add her link. With every State association exhibiting renewed vigor and power, the future is full of promise; and may all who have realized the value of organization spread the good influence in all directions.

